# Effect of comorbid mental pathology on adherence to psychopharmacotherapy in patients with bipolar disorder

**DOI:** 10.1192/j.eurpsy.2026.10700

**Published:** 2026-07-31

**Authors:** S. N. Vasilieva, G. G. Simutkin, S. V. Vladimirova, N. A. Bokhan

**Affiliations:** Mental Health Research Institute, Tomsk, Russian Federation

## Abstract

**Introduction:**

According to Li N.C. et all. (2022) 47.7% of bipolar disorder (BD) patients do not follow physician recommendations. The comorbidity of BD with other mental disorders and somatic diseases significantly reduces patient adherence to psychopharmacotherapy.

**Objectives:**

to evaluate influence of comorbid mental pathology on the level of adherence to prescribed psychopharmacotherapy in BD patients

**Methods:**

Based on the Affective States Department of the clinic of the Mental Health Research Institute of Tomsk NRMC, 61 patients with BD were examined. In the study group, women accounted for 57.4% (n = 35), men 42.6% (n = 26). The median age of male patients was 33 [30; 41] years, female patients - 31 [24; 49 years] year. Within BD, the current mixed episode was detected in 60.7% (n = 37), the hypomanic episode - 1.6% (n = 1), the depressive episode - 37.7% (n = 23).

**Results:**

In the study group, 33 patients (54.1%), along with BD, identified another mental disorder: alcohol dependence, anxiety spectrum disorders, personality disorder. An assessment of adherence based on BD comorbidity with other mental disorders revealed more frequent violations of physician recommendations by patients with this comorbidity, compared to patients with “isolated” BD: 87.9% and 50.0% (Pearson Chi-square: 10.4485, p = 0.001). Correlation analysis confirmed lower adherence in BD patients with other mental disorders (rs = 0.41, p < 0.05).

**Conclusions:**

The presence of comorbid mental pathology in BD negatively affects patients’ adherence to long-term psychopharmacotherapy.

**Disclosure of Interest:**

None Declared

